# Highly potent antioxidant *Olea europaea* L. leaf extract affects carotid and renal haemodynamics in experimental hypertension: The role of oleuropein

**DOI:** 10.17179/excli2017-1002

**Published:** 2018-01-04

**Authors:** Milan Ivanov, Una-Jovana Vajic, Nevena Mihailovic-Stanojevic, Zoran Miloradovic, Djurdjica Jovovic, Jelica Grujic-Milanovic, Danijela Karanovic, Dragana Dekanski

**Affiliations:** 1 Department of Cardiovascular Physiology, Institute for Medical Research, University of Belgrade, Dr Subotića 4, P. O. Box 102, Belgrade, Serbia; 2 Biomedical Research, R & D Institute, Galenika a.d., Pasterova 2, Belgrade, Serbia

**Keywords:** olive leaf extract, oleuropein, vascular resistance, blood pressure, oxidative stress

## Abstract

Haemodynamic alterations in carotid and renal arteries are associated with the severity of target organ damage in patients with hypertension. Dietary habits, such as the Mediterranean diet, regulate blood pressure and oxidative stress, thus reduce the mortality rate due to cardiovascular diseases. In this study, our aim was to evaluate the reducing activity, antioxidant capacity and metal chelating ability of standardized *Olea europaea *L. leaf extract (OLE), and to test its (5, 25, 50 mg/kg) acute *in vivo* effects, as well as oleuropein's (OP, 10 mg/kg) on oxidative stress, carotid, renal and systemic haemodynamic parameters (blood pressure, heart rate, cardiac output, peripheral resistance) in spontaneously hypertensive rats (SHR). OLE has a higher antioxidative capacity than BHT, higher reducing ability than vitamin C, and 23 times lower capacity for metal ion chelation than EDTA. All three doses of OLE, and OP, improved oxidative stress in SHR. OLE5 improved carotid and renal haemodynamics, without significant effects on systemic haemodynamics. Two different mechanisms of antihypertensive responses to OLE were observed, OLE25 was most effective in reducing cardiovascular risks by improving systemic and regional (carotid and renal) haemodynamics, peripheral and regional vascular resistance. OLE50 causes the improvement of blood pressure and cardiac performances, but tends to retain elevated vascular resistance, therefore, reducing the inflow of blood into the brain and kidneys of the SHR. The OP did not alter systemic or regional haemodynamics, suggesting others constituents responsible for changes of cardiac function, as well as carotid and renal haemodynamics in response to OLE50.

## Introduction

In the last decades, numerous studies have reported many relations between nutrition and health. Dietary habits, such as the Mediterranean diet, can be involved in the regulation of blood pressure, decrease of oxidative stress, improvement of lipid profile and reduction of mortality rates due to cardiovascular diseases (Martinez-Gonzalez et al., 2009[23]). *Olea europaea* L. is among the intensively studied plants, which is highly represented in the Mediterranean diet. 

So far, different experimental models have been used with the intention to investigate the hypotensive effect of *Olea europaea* L. leaf extract (OLE). For instance, oral administration of OLE showed a dose-dependent prophylactic effect against the rise in blood pressure induced by L-NAME (Khayyal et al., 2002[18]). Besides, commercial OLE caused a concentration-dependent decrease in systolic left ventricular pressure and heart rate on isolated rabbit hearts (Scheffler et al., 2008[36]). Information is also available regarding its activity on experimental models of genetic induced hypertension after chronic oral administration (Romero et al., 2016[34]). The dual effect of OLE in both reducing blood pressure and improving lipid profile was presented as the result of the clinical trials (Perrinjaquet-Moccetti et al., 2008[32]; Susalit et al., 2011[40]). In one word, cardioprotective properties of olive leaf extract are reflected in its ability to reduce blood pressure, exert an antiarrhythmic effect, and preserve coronary blood flow. These properties were exercised probably due to different mechanisms such as the blockade of beta receptors (Somova et al., 2004[37]), angiotensin-converting enzyme (ACE) inhibition, Ca^2+^ antagonism (Susalit et al., 2011[40]), as well as vasodilatory NO pathways (Miloradović et al., 2013[27]).

Haemodynamic alterations in carotid and renal arteries are associated with the severity of target organ (brain, kidney, heart) damage in patients with hypertension, and Ohta et al. (2008[29]) showed positive correlation between carotid vascular resistance and renal vascular resistance, indicating that an increase in vascular resistance in those vascular beds may develop in parallel. On the other hand, oxidative stress plays a significant role in the pathogenesis of cardiovascular disorders, and reactive oxygen species (ROS) concentration had been shown to be increased in human carotid arteries of older patients, as a consequence of excessive production of superoxide ions by NADPH oxidase (Lucas et al., 2016[22]). Several animal studies have also provided an evidence for increased ROS production in the vascular tissue of hypertensive rats, contributing to the generation and maintenance of hypertension (Vaziri et al., 2002[41]). Recently, we have reported significant beneficial effect of different polyphenol-reach plant products on hypertension-induced alterations of the carotid, but not renal artery haemodynamics (Mihailovic-Stanojevic et al., 2016[25][26]), although these effects were accompanied by a significant reduction of blood pressure and systemic oxidative stress. However, to our knowledge, there is no information available on the effects of OLE or its main constituent, oleuropein (OP) on haemodynamics of these two different vascular beds. Olive oils have been shown to be rich in polyphenols and unsaturated fatty acids that are responsible for the enormous antioxidative capacity of this plant (Ferrara et al., 2000[13]; Krzeminski et al., 2003[20]; Žaneti and Gugi, 2006[42]). In addition, antioxidant properties of the OLE that lead to reduction of lipid peroxide accumulation in blood and other target organs, have been attributed to the high content of OP and its derivative hydroxytyrosol (Alirezaei et al., 2012[1]; Suanarunsawat et al., 2011[38]). Beside their antioxidative potential, these compounds showed antihypertensive and hypocholesterolemic properties and they have been effective in lowering triglycerides as well. 

The first aim of the present study was to evaluate the reducing activity, antioxidant capacity and metal chelating ability of standardized, OP-rich OLE. The second goal was to evaluate the existence of its dose-dependent influence on systemic and regional haemodynamics, as well as the mechanism of antihypertensive action in spontaneously hypertensive rats (SHR). Lipid profile and lipid peroxidation were also investigated in this experimental model of essential hypertension. In addition, we compared the observed effects with those caused by OP alone. 

## Materials and Methods

### Chemicals

Olive leaf extract EFLA^®^ 943, standardized to 16-24 % of OP, was purchased from Frutarom Switzerland Ltd. (Wadenswil, Switzerland). The extract was manufactured from the dried leaves of *Olea europaea* L., applying an ethanol (80 % m/m) extraction procedure. The crude extract was dried after a patented filtration process (EFLA^®^ Hyperpure). Oleuropein content, stability and microbiological purity were confirmed by the manufacturer. According to the Certificate of Analysis provided by Frutarom, the batch used in our experiment contained 17 % m/m of OP and 40.5 % m/m of total polyphenols. Residual solvent ethanol was 0.1 % m/m only. Oleuropein (purity ≥ 90 %) was obtained from Extrasynthese (Geney, France). All other reagents were obtained from Sigma-Aldrich Chemical Co., USA, unless otherwise stated.

### Ferric reducing antioxidant power of OLE

The antioxidant potential of OLE was determined using ferric reducing antioxidant power assay (FRAP) (Benzie and Strain, 1996[4]). It is based on the change in absorbance at 593 nm as a result of the reduction of ferric to ferrous ion and consequent formation of a Fe(II)-tripyridyl triazine. 

### Trolox Equivalent Antioxidant Capacity of OLE

Another method used for determination of antioxidant capacity of OLE was Trolox equivalent antioxidant capacity assay (TEAC). This assay is based on the ability of antioxidants to reduce the ABTS• +, a stable radical, formed in the reaction of ABTS (2,2'-azinobis (3-ethylbenzothiozoline-6-sulfonic acid)) and potassium persulfate (Krishnaiah et al., 2011[19]). This reduction results in a decrease in absorbance at 734 nm. The assay was done according to a previously described method (Re et al., 1999[33]). 

### Metal chelating ability of OLE

Metal chelating ability was determined using a method based on a decrease in absorbance of Fe(II)-ferrozine complex (Dinis et al., 1994[9]). This method was done with slight modifications. 450 μL of 1.5 mM FeCl_2_ and 1.8 mL of 385 μM ferrozine were added to 50 μL of OLE solution. The mixture was vigorously vortexed and after 10 min, the absorbance was measured at 562 nm. All measurements were performed in triplicate. EDTA (ethylenediaminetetraacetic acid), a potent Fe^2+^ ion chelator, was used as positive control. The result was expressed as an EC value (mg/mL, the half maximal effective concentration of OLE or EDTA).

### Animals and experimental protocol

The experimental protocol was approved by the Ethic Committee of the Institute for Medical Research, University of Belgrade, Serbia (No. 0316-1/11), according to the National Law on Animal Welfare ("Službeni Glasnik" no. 41/09) that is consistent with guidelines for animal research and principles of the European Convention for the Protection of Vertebrate Animals Used for Experimental and Other Purposes (Official Daily N. L 358/1-358/6, 18, December 1986) and Directive on the protection of animals used for scientific purposes (Directive 2010/63/EU of the European Parliament and of the Council, 22.9.2010.).

We used 24 weeks old male spontaneously hypertensive rats (SHR), weighing about 300 g, which were bred at the Institute for Medical Research, University of Belgrade, Serbia. The rats were housed 4 in a cage under constant environmental conditions (20-24 °C; 12 h light-dark cycle), and fed *ad libitum* with a standard chow for laboratory rats (Veterinarski zavod, Subotica, Serbia).

For the evaluation of dose-dependent effects of OLE on systemic haemodynamic parameters before and after treatment, SHR were randomly divided into three groups: OLE5 (n=7), OLE25 (n=7), OLE50 (n=7) which received bolus of OLE 5, 25, and 50 mg/kg b.w., dissolved in 0.2 ml saline. The highest dose of OLE used in this experiment (50 mg/kg) was the same, the highest dose used previously in the study in which Zarzuelo et al. (1991[43]) were found that the decoction of olive leaves showed spasmolytic activity against phenylephrine-induced contractions in isolated rat aorta (Zarzuelo et al., 1991[43]). The investigated dose of OP, 10 mg/kg (dissolved in 0.2 ml saline, group OP10, n=7) corresponded to its content in OLE50 (app 20 % m/m). The control SHR group received 0.2 ml saline (n=7). 

Before surgical procedure, all rats were weighed and anesthetized with 35 mg/kg of sodium pentobarbital. 

### Systemic haemodynamic measurements 

Systemic haemodynamic parameters were measured in anesthetized rats by a direct method. A femoral artery was catheterized with polyethylene tubing (PE-50, Clay-Adams Parsippany, NY, USA), and connected to a physiological data acquisition system (9800TCR Cardiomax III-TCR, Columbus, OH, USA). A jugular vein was cannulated with PE-50 for the injection of cold saline. The left carotid artery was catheterized with PE-50 tubing and attached to a thermo sensor, which was coupled to the Cardiomax III for the determination of cardiac output (CO). The other end of the thermocouple was placed in cold saline. Following 20 min for stabilization after surgery, cold saline (0.2 ml) was supplied through the jugular vein and systolic pressure (SAP), diastolic (DAP), mean arterial pressure (MAP), heart rate (HR), and CO were recorded before and after saline, OLE or OP treatment. Total peripheral vascular resistance (TPVR) was calculated from MAP and CO and expressed as mmHg x min x kg/ml. Pulse pressure (PP) is calculated as the difference between the systolic and diastolic arterial pressure readings and it is measured in millimeters of mercury (mmHg).

### Regional haemodynamic measurements

For the carotid blood flow (CBF) measurement, the left carotid artery was gently separated. An ultrasonic flow probe (1RB, internal diameter = 1 mm) was placed around the artery and CBF had been registered using a Transonic T106 Small Animal Flowmeter (Transonic System Inc., Ithaca, NY, USA). After abdominal incision, left renal artery preparation was utilized and renal blood flow (RBF) was recorded. Vascular resistance in these two vascular beds - carotid and renal arteries (CVR and RVR) was calculated by dividing MAP with total blood flow through a respective blood vessel, normalized for the body weight and expressed as mmHg x min x kg/ml. 

### Sample collection

After haemodynamic measurements, blood samples were taken by puncture of the abdominal aorta into the tubes containing lithium-heparin (Li-heparin, Sigma, USA) as an anticoagulant and centrifuged at 4000 rpm, 4 °C for 20 minutes. Plasma samples were stored on -20 °C until assayed for lipid peroxidation and lipid profile.

### Lipid peroxidation

In order to determine the effects of different OLE doses, as well as OP on the degree of oxidative stress in the circulation, plasma lipid peroxide levels were estimated by assaying thiobarbituric acid reactive substances (TBARS) at 540 nm using 2-thiobarbituric acid (2,6-dihydrooxypyrimidine-2-thiol; TBA, Acros, Organic). An extinction coefficient of 156000 M^-1^ cm^-1^ was used for calculation (Ohkawa et al., 1979[28]). To evaluate the rate of TBARS released in vascular beds of interest, carotid and renal arteries, plasma TBARS values were normalized to respective blood flows (CBF and RBF) and expressed as nmol/min/ kg b.w.

### Lipid profile determination

Total cholesterol, high-density lipoprotein cholesterol (HDL-C) and triglyceride levels in plasma were measured using an automatic COBAS INTEGRA 400 plus (Hoffmann-La Roche, Germany) analyzer. 

### Statistical analysis

The results are shown as the mean with the standard error of the mean. We used the one-tailed Student's t-test for two-samples of equal variance for parameters which were analyzed before and after treatment and p < 0.05 was considered as notable (Microsoft Excel 2010). For statistical analysis of other parameters one-way analysis of variance (ANOVA) was applied. When the ANOVA results were significant, LSD (least significant difference) was performed as a *post hoc* multiple comparison (Statistics for Windows). P values < 0.05 were considered significant.

## Results

### In vitro antioxidant capacity assessment 

Estimation of antioxidative potential of OLE was made by ferric reducing antioxidant power (FRAP method), by assessment of free radical scavenging activity (TEAC), and metal chelating ability, and the results are shown in Table 1(Tab. 1). FRAP of OLE was 22 times higher compared to BHT, and it was almost 50% more effective than vitamin C. Similarly, TEAC assay showed that the antiradical capacity of OLE was 2 times greater than the antiradical capacity of BHT, but 4 times lower than the antiradical capacity of vitamin C. Metal chelating ability of OLE was compared to EDTA, and this analysis showed that OLE has 23 times lower capacity for metal ion chelation than EDTA.

### Haemodynamic measurements

**Figure 1:**
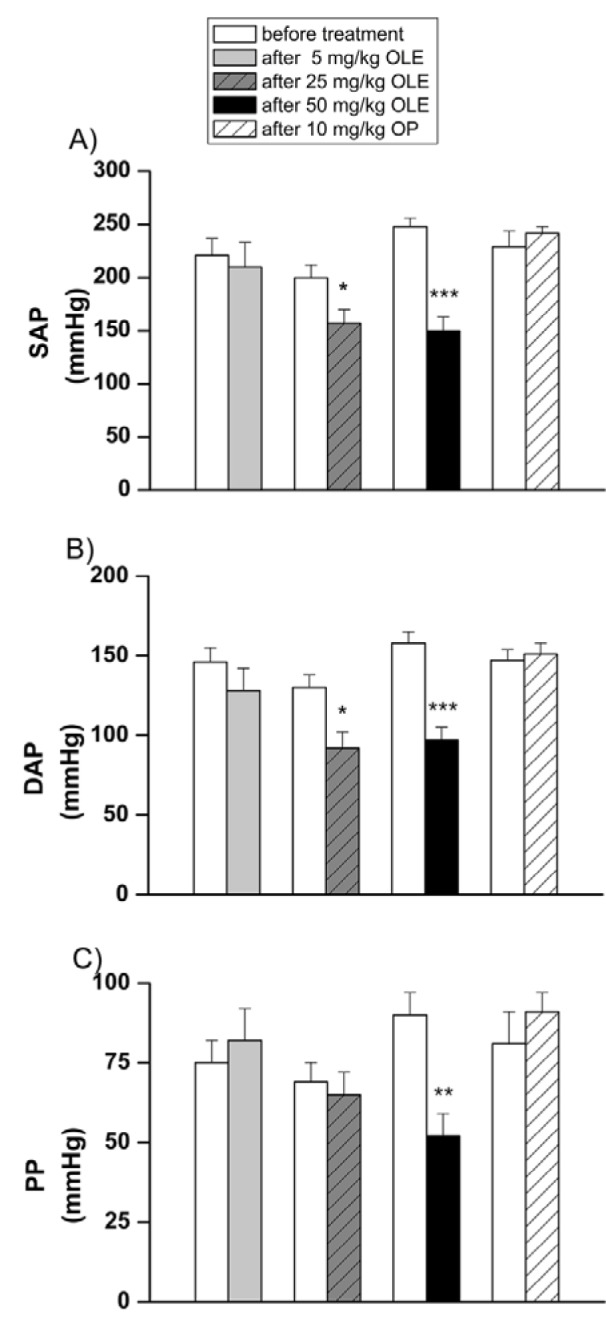
Systolic (SAP), diastolic (DAP) and pulse pressure (PP) before and after treatments OLE-* Olea europaea* L. leaf extract, OP- oleuropein, *p < 0.05, **p < 0.01, ***p < 0.001 the significant difference between values before and after treatment

**Figure 2:**
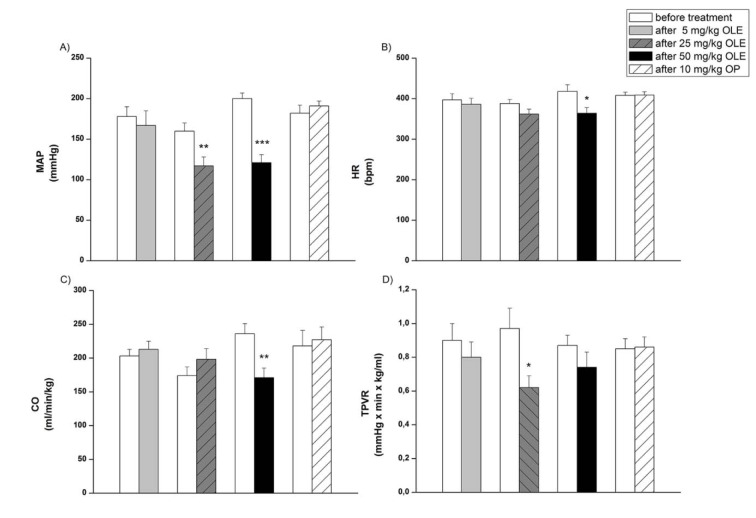
Mean arterial pressure (MAP), heart rate (HR), cardiac output CO and total peripheral vascular resistance (TPVR) before and after treatments OLE-* Olea europaea* L. leaf extract, OP- oleuropein. *p < 0.05, **p < 0.01, ***p < 0.001 the significant difference between values before and after treatment

Treatment with 5 mg/kg OLE had no effect on systemic haemodynamic parameters of SHR (Figure 1(Fig. 1) and 2(Fig. 2)).

**Figure 3:**
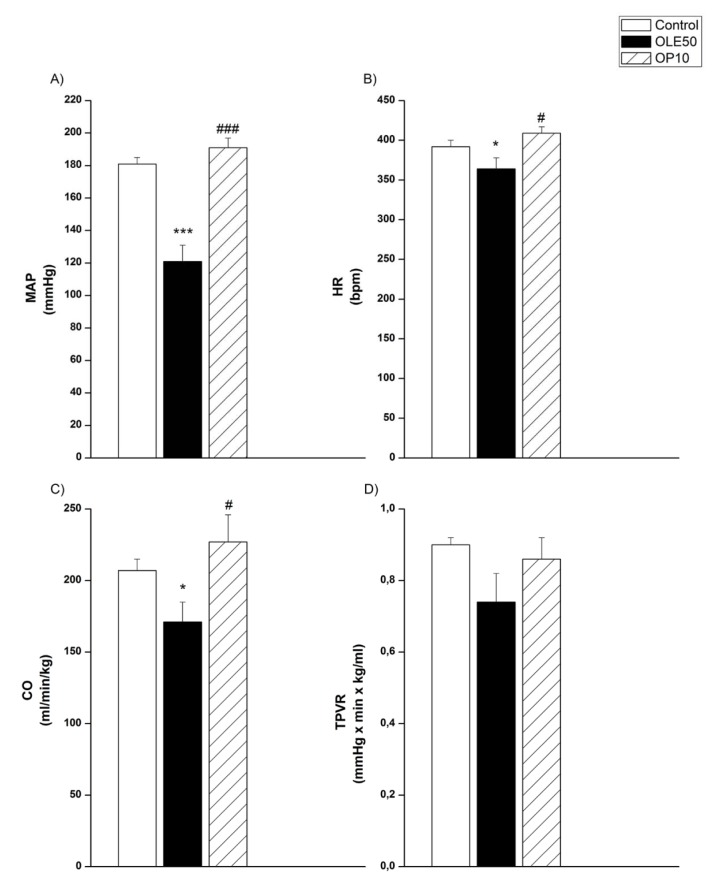
Mean arterial pressure (MAP), heart rate (HR), cardiac output (CO) and total peripheral vascular resistance (TPVR) OLE-* Olea europaea* L. leaf extract, OP- oleuropein, *p < 0.05, ***p < 0.001 the significant difference vs.control. #p < 0.05, ###p < 0.001 the significant difference vs. OLE50

SAP, DAP (Figure 1A, 1B(Fig. 1)) and MAP (Figure 2A(Fig. 2)) were significantly decreased after the application of both higher doses of OLE, 25 and 50 mg/kg, compared to values obtained before treatment. Pulse pressure was significantly lower only after treatment with a high dose (50 mg/kg) of OLE (Figure 1C(Fig. 1)). In OLE50 group, HR and CO were also significantly decreased compared to the initial values (Figure 2B, 2C(Fig. 2)), while in OLE25 group significant reduction of TPVR was noticed (Figure 2D(Fig. 2)). We further examined the contribution of OP to the OLE50-induced changes of systemic haemodynamic parameters (Figure 3(Fig. 3)) in SHR. The reduction of MAP, HR and CO values that have been obtained in OLE50, were absent in OP10 group (Figure 3A, 3B, 3C(Fig. 3)) while there were no differences among TPVR values (Figure 3D(Fig. 3)).

**Figure 4:**
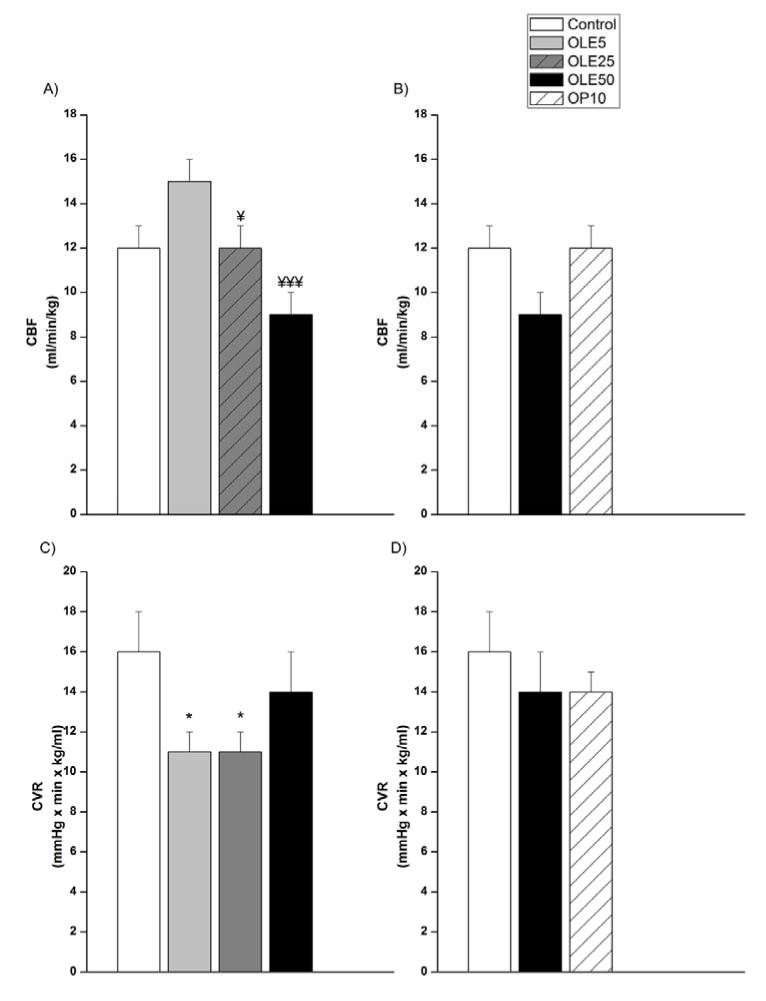
Carotid blood flow and carotid vascular resistance of experimental groups OLE-* Olea europaea* L. leaf extract, OP- oleuropein, *p < 0.05, the significant difference vs.control. ^¥^p < 0.05, ^¥¥^p < 0.01, the significant difference vs. OLE5

Treatment with 5 mg/kg of OLE slightly improved CBF (Figure 4A(Fig. 4), p=0.07), and significantly decreased CVR compared to the control group (Figure 4C(Fig. 4), p < 0.05). The dose of 25 mg/kg of OLE significantly reduced CVR (p < 0.05), too. Comparison between OLE treated groups show that OLE5 significantly elevates CBF in relation to OLE25 and OLE50 (p < 0.05, and p < 0.001). An analysis of the effects of OP10 in relation to OLE50 and control (Figure 4B, 4D(Fig. 4)) has shown that there was no significant difference in CBF or CVR between these groups, although the decrease of CBF in OLE50 was numerically, but not statistically different in respect to both, the control and OP10 groups (Figure 4B(Fig. 4), p=0.09).

**Figure 5:**
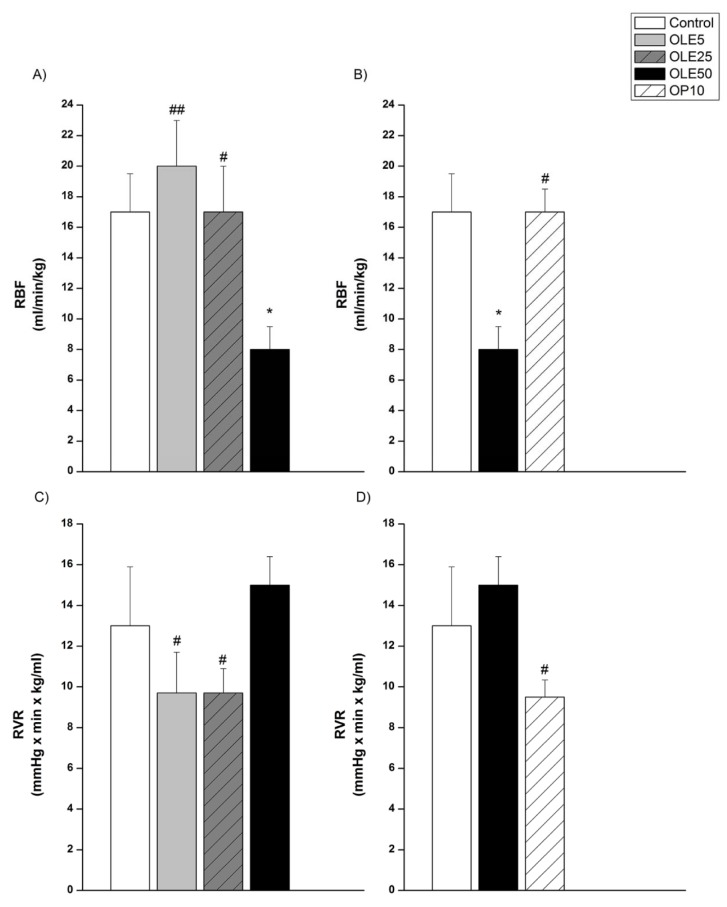
Renal blood flow and renal vascular resistance of experimental groups OLE-* Olea europaea* L. leaf extract, OP- oleuropein, *p < 0.05 the significant difference vs.control. #p < 0.05, ##p < 0.01 the significant difference vs. OLE50

Effects of all three doses of OLE on RBF and RVR, as well as the comparison of the effects of OP10, OLE50 and control values of these parameters are presented in Figure 5(Fig. 5). Treatment with the highest dose of OLE (50 mg/kg) significantly decreased RBF, compared to control group (p<0.05), and other OLE treated groups (p<0.01 vs. OLE5, p<0.05 vs. OLE25, Figure 5A(Fig. 5)), as well as compared to OP10 (p<0.05, Figure 5B(Fig. 5)). Both, OLE5 and OLE25 significantly reduced RVR in comparison to the value noticed in OLE50 (p<0.05, Figure 5C(Fig. 5)), although these reductions did not significantly change with respect to the control group. RVR obtained for the control, OLE50 and OP groups (Figure 5D(Fig. 5)) showed the highest level in OLE50 group, and it was significantly reduced in OP10 group (p<0.05).

### Lipid peroxidation and lipid profile

**Figure 6:**
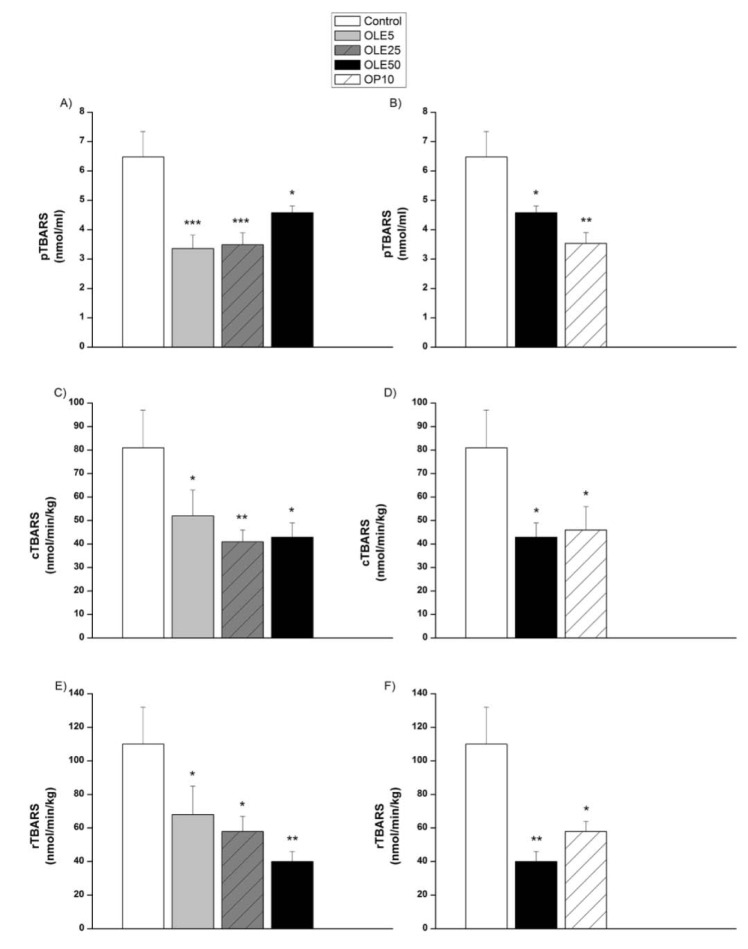
Plasma (p) TBARS concentration and the release of TBARS in carotid (c) and renal (r) arteries of experimental groups OLE- Olea europaea L. leaf extract, OP- oleuropein, *p < 0.05, **p < 0.01, ***p < 0.001 the significant difference vs.control

All treated groups showed significantly lower levels of plasma TBARS concentration compared to the control group (OLE5 and OLE25, vs. control, p < 0.001, OLE50 vs. control, p < 0.01, Figure 6A(Fig. 6); OP10, vs. control, p < 0.001, Figure 6B(Fig. 6)). The release of TBARS was significantly reduced in both, carotid (Figure 6C, 6D(Fig. 6)) as well as renal (Figure 6E, 6F(Fig. 6)) arteries, in all treated groups compared to the control group. 

**Figure 7:**
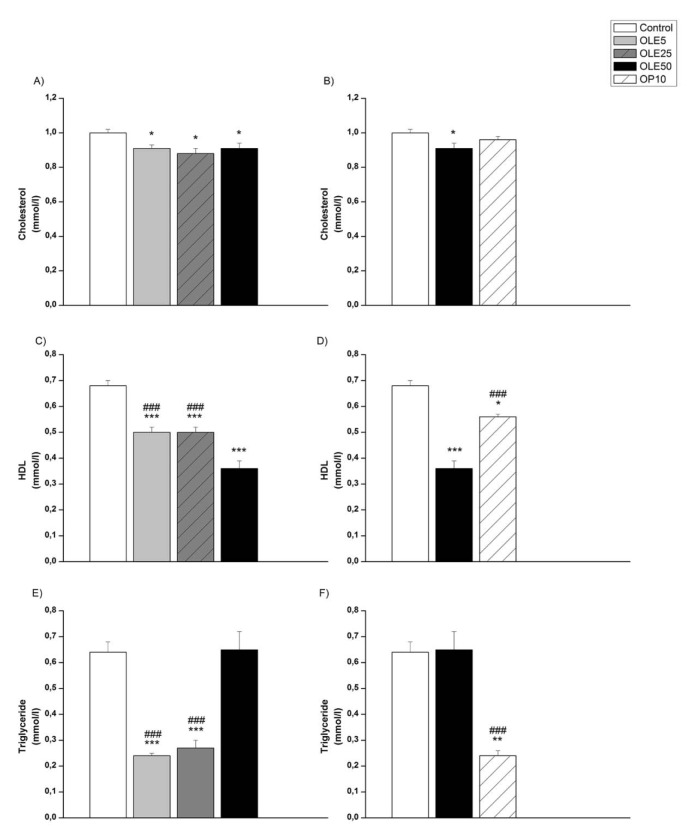
Lipid profile of experimental groups OLE-* Olea europaea* L. leaf extract, OP- oleuropein, *p < 0.05, **p < 0.01, ***p < 0.001 the significant difference vs. control. ###p < 0.001 the significant difference vs. OLE50

Lipid profile's data are shown in Figure 7(Fig. 7). Treatment with all dosages of OLE (Figure 7A(Fig. 7)) significantly decreased the values of total cholesterol (p < 0.05), but treatment with 10 mg/kg of OP showed no effect (Figure 7B(Fig. 7)). In comparison with the control group, all treatments significantly decreased HDL (OLE5, OLE25 and OLE50 vs. control p < 0.001, Figure 7C(Fig. 7); OP10 vs. control p < 0.05, Figure 7D(Fig. 7)) with a considerable decrease in the OLE group which received 50 mg/kg. Moreover, in this group, HDL levels were significantly lower than in OLE5, OLE25, and OP10 (p < 0.001).

Groups treated with 5 mg/kg and 25 mg/ kg of OLE (Figure 7E(Fig. 7)), and 10 mg/kg of OP (Figure 7F(Fig. 7)) showed significantly lower level of triglycerides in plasma compared to the control group (p < 0.001), as well as compared to OLE50 group (p < 0.001). There was no difference in triglyceride levels between the control group and the group treated with 50 mg/kg of OLE.

See also the Supplementary data.

## Discussion

In this study, we showed that OLE has a higher antioxidative capacity than BHT, and higher reducing ability than vitamin C, a potent natural antioxidant. These data were in accordance with previously determined radical scavenging activity of OLE (Čabarkapa et al., 2017[7]), using the 2,2-diphenyl-1-picrylhydrazyl (DPPH·). Namely, the values for the effective concentration (50 % inhibition) that they obtained indicate a moderate antioxidant capacity of the extract (Čabarkapa et al., 2017[7]). Furthermore, if we compare the OLE ability to chelate metal ions with chelation capacity of other evaluated natural extracts (Bourgou et al., 2008[5]; Ebrahimzadeh et al., 2008[10]; Liu et al., 2008[21]), we assume that this extract can prevent vascular oxidative stress caused by elevated ROS production, thus reduce the cardiovascular risks in SHR. In addition, this effect is probably coming from OP (the most abundant phenolic compound in OLE), because of its ability to chelate metal ions, such as Cu and Fe, which catalyze free radical generation reactions (Omar, 2010[30]; Suchal et al., 2016[39]).

In the second part of the study, we showed the existence of two different mechanisms of the antihypertensive response to OLE in SHR dependent on the dose that was applied. Namely, when SHR was administered with 25 mg/kg OLE, it affected vascular mechanisms that regulate the resistance in a systemic circulation, resulting in an induction of significant reduction of TPVR, and therefore SAP, DAP, and MAP. Contrary, changes involving cardiac mechanisms which reflected on HR and CO occurred after application of OLE in a dose of 50 mg/kg. The heart rate in OLE50 group was significantly reduced, leading to the decrease of CO and also to the decrease of blood pressure. On the other hand, in OLE25 group, blood pressure was also decreased, but at the expense of significant peripheral vasodilation (decreased total peripheral resistance), without altering the CO or HR. Previously, we demonstrated that acute oral administration of 2 g/kg of OLE reduces SAP in SHR (Dekanski et al., 2014[8]), and that 30 min duration of antihypertensive action of OLE (50 mg/kg) is NO-independent, whereby retaining antihypertensive effect occurs even after the blocked synthesis of NO, indicating that OLE in a dose of 50 mg/kg achieves blood pressure-lowering by mechanisms other than NO (Miloradović et al., 2013[27]). The ACE inhibitory activity of OLE, concurrent with the calcium channel blocking activity, as a result of the synergistic activity of OP and other components of OLE, was assumed by Susalit et al. (2011[40]) in the clinical study in which the antihypertensive effect of OLE and captopril were compared. However, the study of a cardiotonic and antiarrhythmic effects of olive derivatives has shown that the effect of oleanolic acid (10 mg/kg) was similar to the effect of propranolol, a nonselective beta blocker (2 mg/kg) (Somova et al., 2004[37]). The fact that in our study, blood pressure was normalized after the administration of OLE (25 and 50 mg/kg), but not the OP (10 mg/kg) alone, led us to a conclusion that other components presented in the extract could be responsible for OLE-induced systemic haemodynamic alterations, or possibly, the synergism between the OP and those other components are important for such response. Our finding that the OP does not contribute to blood pressure lowering effect of OLE is in accordance with previous findings, which showed OP (10 and 20 mg/kg during the 3 or 6 weeks) had no effects on blood pressure in rabbits with myocardial infarction (Andreadou et al., 2007[3]), or did not show antiarrhythmic effects in isolated rat heart (Esmailidehaj et al., 2012[12]). 

Pulse pressure is an independent risk factor for the development of cardiovascular diseases. Previously, it has been shown that alpha/beta blocker in combination with ACEI reduces the cardiovascular risk due to a decrease in pulse pressure (Jovanovic et al., 2009[17]). The present results also demonstrate a significant reduction in pulse pressure in SHR after a dose of 50 mg/kg OLE during the acute application, which may be explained by the previously mentioned ACEI activity, or the possible beta blockers effects of the extract (Susalit et al., 2011[40]).

Earlier, we showed up to 3 fold higher CVR in SHR than in their normotensive counterparts, and that this could be significantly improved by acute polyphenol reach plant extract treatment (Mihailovic-Stanojevic et al., 2016[25]). Here, the lower doses of the OLE, 5 and 25 mg/kg, also led to a significant reduction of CVR of SHR, which in the case of a dose of 25 mg/kg was concomitant with a significant decrease of MAP. The reduction of CVR was absent after application of a dose of 50 mg/kg OLE and 10 mg/kg OP, further confirming the contribution of vascular mechanisms (involved in the regulation of systemic and regional vascular resistance) in a fall of blood pressure in the group OLE25.

Haemodynamic changes in the kidney have a significant role in the maintenance of hypertension. Back in 1986, Ando and co-workers showed high positive correlation between renal resistance and MAP in patients with essential hypertension. Specifically, the treatment with ACEI, captopril, has led to a significant decrease in the renal resistance and mean arterial blood pressure, while the blood flow through the renal artery was slightly, but not significantly increased (Ando et al., 1986[2]). Our results of renal haemodynamics are in accordance with the before mentioned, because, the application of an OLE, as well as OP, for which it has long been established to have ACE inhibitory activity (Hansen et al., 1996[15]), had no significant effect on blood flow through the renal artery. On the other hand, doses of 5 and 25 mg/kg OLE, and 10 mg/kg of OP lead to a reduction in the resistance of the vascular bed of hypertensive rats. This decrease in RVR in SHR treated with lower doses of OLE, together with a statistically significant reduction in the CVR, significantly contributes to the reduction of TPVR and the normalization of blood pressure in our experimental model of essential hypertension.

In our previous study, we already showed that hypertension is followed by increased oxidative stress (Miloradović et al., 2013[27]). After all OLE treatments (5, 25, and 50 mg/kg), in the present study, there was a significant reduction of plasma lipid peroxidation in SHR, as well as in the group treated with 10 mg/kg of OP alone, in comparison to the control group. It is well known that OLE has potent in vitro (Hayes et al., 2011[16]), as well as, in vivo antioxidant activity due to the synergy between flavonoids, oleuropeosides and substituted phenols (El and Karakaya, 2009[11]). Since the oxidative stress in the circulation of SHR, in our study, showed the same trend of decreasing after administration of all three doses of OLE, it can be assumed that the antioxidant response is not dose-dependent, and since it is almost the same in the OP group compared to OLE50, the OP may be held responsible for this effect. In accordance with our results is the study of long-term effects of OP which shows that OP (oral dose of 15 mg/kg) reduces the oxidative stress, the level of TBARS, total cholesterol, and triglycerides in the model of the ethanol-induced hepatotoxicity by increasing the activity of antioxidant enzymes (Alirezaei et al., 2012[1]). 

Acute application of OLE in doses of 5, 25 and 50 mg/kg, led to a reduction in the total cholesterol level, which points to the hypocholesterolemic effect of OLE. Treatment of SHR with 5 and 25 mg/kg of OLE, has led to a statistically significant decrease in triglyceride compared to control and OLE50 group. In rats with streptozotocin-induced diabetes (Ghanema and Sadek, 2012[14]), the group treated with the OLE showed a reduction of the level of total cholesterol and triglycerides, but also the level of TBARS as a result of reduced oxidative stress due to increased concentrations of antioxidative enzymes. In our experiment, HDL was also decreased by all doses of OLE compared to the control group, but most pronounced in the group administered with a dose of 50 mg/kg of OLE. The triglyceride level was higher in this group compared to OP10 group. A significant reduction in the level of HDL and the increase in levels of triglycerides (Perona et al., 2004[31]), has been previously observed in elderly hypertensive subjects who used olive oil in their diet and were treated with beta-blockers and diuretics. Given the fact that beta blockers tend to increase triglyceride levels (direct reduction of their catabolism), while reducing the level of HDL (Brook, 2000[6]), and with our assumption that high doses of OLE exhibit their antihypertensive and cardioprotective properties, among other things, by blockade of the beta-adrenergic receptor, it can be concluded that beta blockers characteristic of the components present in the 50 mg/kg OLE, different from the OP, are responsible for a significant increase in the concentration of triglycerides and the reduction of the amount of HDL in our study. However, there are opposing opinions about the effects of olive rich diet on high-density lipoproteins (HDL). Some studies showed that this diet can lead to a decrease of HDL due to a higher concentration of unsaturated fatty acids, polyphenols, and squalen (Mensink and Katan, 1992[24]; Perona et al., 2004[31]). On the other hand, a diet rich in virgin olive oil can increase plasma HDL levels (Rondanelli et al., 2016[35]). In a study comparing the effects of olive leaf extract (500 mg twice daily during 8 weeks, ~ 12.5 mg/kg/day) and captopril on blood pressure and lipid profile in patients with the stage 1 hypertension (Susalit et al., 2011[40]), in addition to the antihypertensive activity, there was a significant reduction in total cholesterol and triglyceride levels after administration of OLE, compared with the group treated with captopril, in which there was no change of lipid status. 

In conclusion, the lowest used dose of OLE, 5 mg/kg, successfully abolished oxidative stress and showed a trend to normalize regional (carotid and renal) haemodynamics, but was insufficient to reduce blood pressure. Medium dose, 25 mg/kg, was revealed as the most effective in reducing cardiovascular risks by improving systemic and regional haemodynamics, oxidative stress and lipid profile. Furthermore, the dose of 50 mg/kg OLE causes the improvement of blood pressure, antioxidative defense and cardiac performances in such acute study, but it tends to retain elevated vascular resistance, therefore reducing the inflow of blood into the brain and kidneys of the SHR. The main constituent of this standardized extract, OP alone, failed to ameliorate systemic haemodynamics, indicating that other components of OLE are responsible for changes of cardiac function in SHR. The antioxidative potential, but also the ability of OP to improve the lipid profile, unlike the OLE50, enables it to act as cardioprotectant.

## Acknowledgements

This work was supported by grants from the Ministry of Education, Science and Technological Development of the Republic of Serbia (No OI 175096 and III 46010). The authors gratefully acknowledge the professional English language assistance provided to us by Vladana Ivanov, MA in English language.

## Supplementary Material

Supplementary data

## Figures and Tables

**Table 1:**
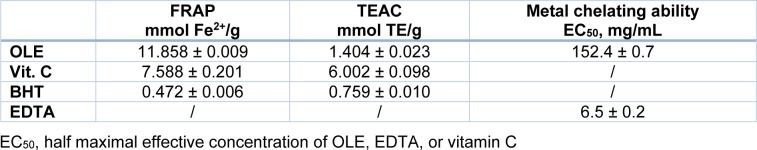
Ferric reducing antioxidant power (FRAP), Trolox equivalent antioxidant capacity (TEAC), and the metal chelating ability of *Olea europaea* L. leaves extract (OLE)
